# Applicability of a modified EFAST protocol (r-EFAST) to evaluate hemodynamically unstable patients after percutaneous cardiac intervention

**DOI:** 10.1186/s13089-017-0070-3

**Published:** 2017-06-12

**Authors:** José Luis Vázquez Martínez, Kary Leonisa Quiñones Coneo, Tomas Villen Villegas, María Sánchez Porras, Cesar Pérez-Caballero Macarrón, Ana Coca Pérez, Luis Fernandez Pineda

**Affiliations:** 1 Pediatric Intensive Care Unit, Ramón y Cajal University Hospital, Madrid, Spain; 2 Pediatric Intensive Care Unit, Crta Colmenar Viejo 9100, 28034 Madrid, Spain; 3 Emergency Department, Ramón y Cajal University Hospital, Madrid, Spain; 4 Pediatric Cardiology Department, Ramón y Cajal University Hospital, Madrid, Spain

**Keywords:** Congenital heart defect, Cardiac catheterization, Retroperitoneal space, Hematoma

## Abstract

Percutaneous cardiac intervention is an invasive diagnostic and therapeutic technique which carries a significant complication rate. Although the usefulness of EFAST protocol is widely recognised, this paper will attempt to explore a modified approach involving a focused examination on the retroperitoneal (r-EFAST). We have provided examples of 3 cases where r-EFAST was used to detect retroperitoneal bleeding in critical situations.

## Background

Ultrasound is widely regarded as being an accurate and reliable procedure to evaluate patients who have suffered an acute thoraco-abdominal trauma. Developed in late 1990s [1], the Focused Assessment with Sonography for Trauma (FAST) is one of the first ultrasound protocols for the rapid detection of free peritoneal and/or pericardial fluid. The term “Extended FAST” (EFAST) was adapted during the following decade [2] to include the addition of thoracic ultrasound views, encompassing a broader scope for diagnosis of other possible associated injuries, such as hemothorax or pneumothorax. Since its clinical implementation, EFAST has proven to be more reliable than the physical examination [3].

With the continued advancement of imaging equipment and surgical instruments, the application of interventional radiology techniques is becoming more widespread in the diagnostic and therapeutic management of vascular and cardiac diseases. As a result, however, complications related to this procedure have proportionally increased [4, 5]. Percutaneous cardiac interventions have been associated with the following complications; retroperitoneal hematoma, renal injury, aortic wall hematoma, inferior epigastric injury or local vascular complications (acute thrombosis, distal embolization, dissection, poorly controlled bleeding, pseudoaneurysm or arteriovenous fistula). Retroperitoneal bleeding is one of the more serious complications that can occur as the retroperitoneal space has the capacity to harbour large volumes of blood with no clinical signs and symptoms until hypovolemia occurs late in the clinical course [6]. Complications tend to occur in up to 8% of cardiac catheterization, being more frequently associated with therapeutic rather than diagnostic procedures (15.1% vs. 4.6%). The incidence of non-puncture related bleeding ranges from 0.9 to 1.2%, with the retroperitoneal hematoma being the most common finding (0.12–0.5%). Despite its unfamiliar etiology, the hematomas origin might be spontaneous or iatrogenic, which could be secondary to endovascular trauma or the use of anticoagulation drugs during the procedure. Risk factors predisposing to retroperitoneal haemorrhage after percutaneous transfemoral cardiac intervention are the following: the female sex, low platelet count, sheath removal protocol, the use of hydrophilic guide wire [7] and post-procedure anticoagulation, presenting the greatest risk [8].

The purpose of this paper is to explore the usefulness of a modified EFAST protocol (r-EFAST), including the bedside retroperitoneal clinician-performed assessment, to detect retroperitoneal hematoma or renal parenchymal injury in hemodynamically unstable patients after a percutaneous cardiac intervention without any suspected complications during the procedure in the catheterization laboratory.

## Cases presentation

**Fig. 1:**
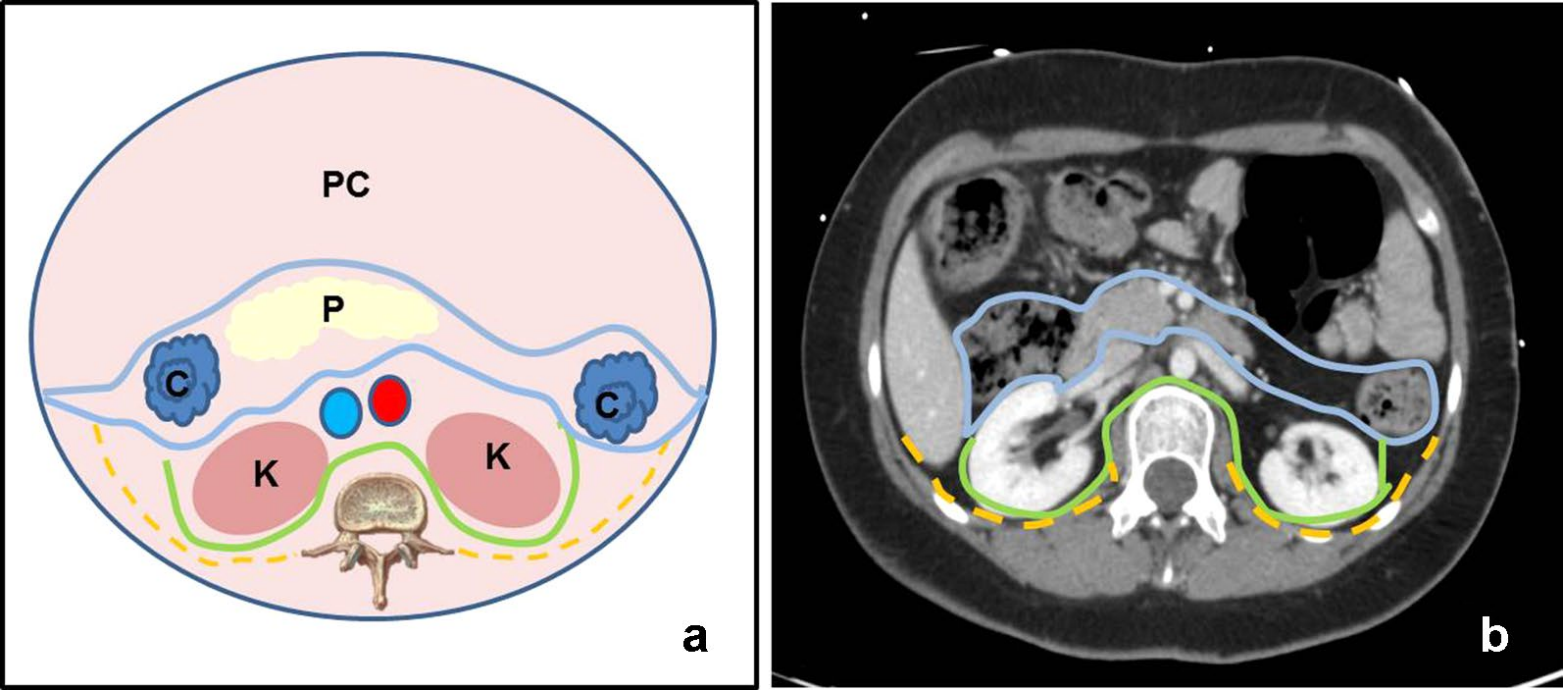
Retroperitoneal space anatomy. **a** Diagram shows the anterior pararenal space (*blue line*), the perirenal space (*green line*) and the posterior pararenal space (*yellow line*). Kidney (K), pancreas (P), colon (C) and peritoneal cavity (PC) **b** Perirenal and pararenal spaces in CT Scan with intravenous contrast

**Fig. 2:**
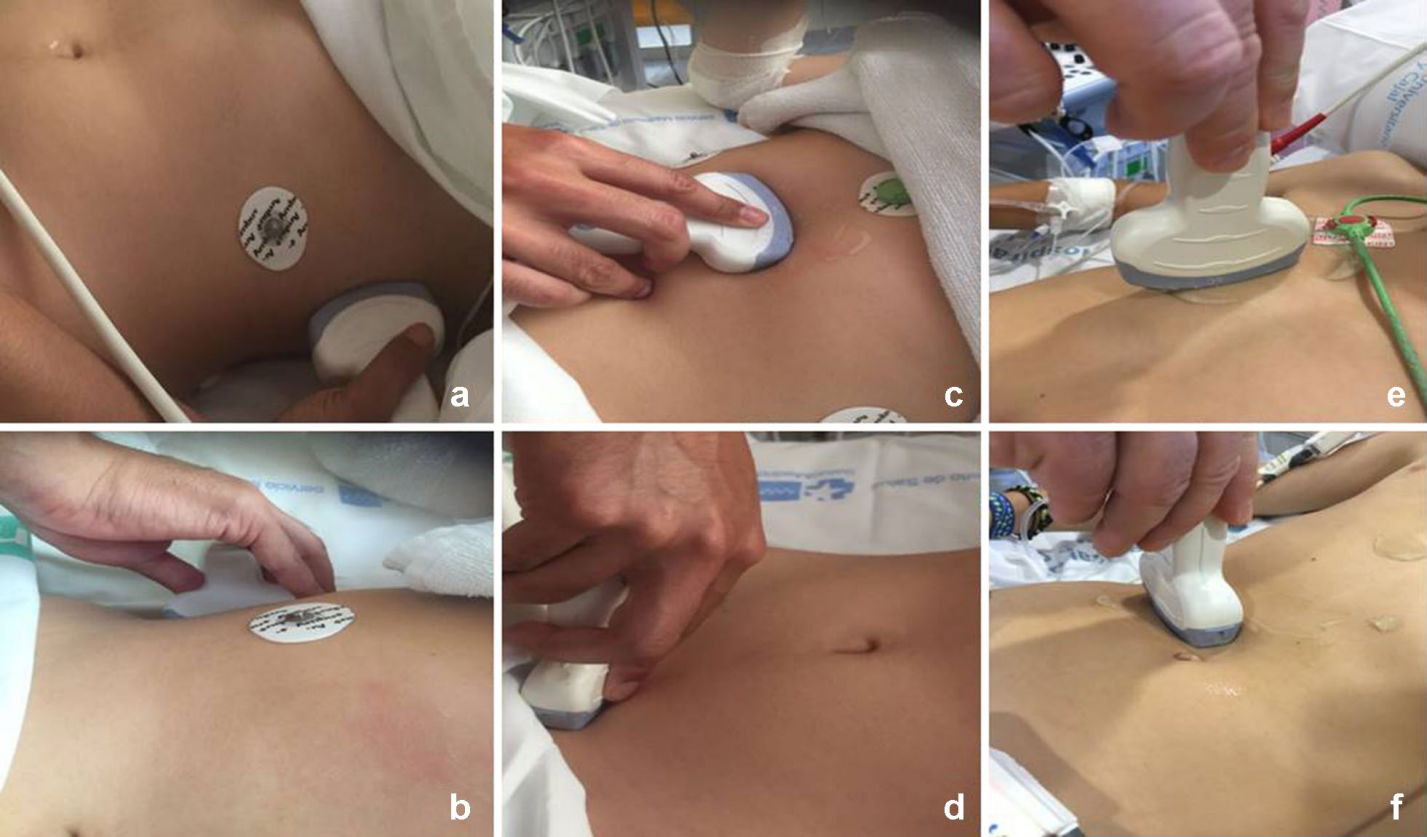
r-EFAST views: **a** *Upper left* abdominal quadrant. **b** *Upper right* abdominal quadrant. **c** Pericardial. **d** Pelvis. **e** Anterior thoracic. **f** Transverse epigastric

We will present the cases of three patients with congenital heart disease without a previous medical history of hemorrhagic diathesis, family history of bleeding or lumbar trauma, who have been diagnosed with a retroperitoneal hematoma after percutaneous cardiac intervention using the r-EFAST protocol. r-EFAST is a bedside, rapid ultrasound protocol used to detect retroperitoneal hematoma or parenchymal renal injury. Once the standard EFAST has been performed in the supine position (scanning the upper right and left abdominal quadrants, pericardial, pelvis and anterior thoracic views), patients are adjusted, to a moderate degree, in a lateral decubitus position. This provides a clearer scan of the retroperitoneal space (Fig. 1), by sliding the probe for the upper right and left postero-lateral thoraco-abdominal junction zone, although the pararenal spaces are best shown transverse epigastric view (Fig. 2).

The examinations were carried out using a TOSHIBA with convex array transducer 3.5–5 MHz and a linear array transducer 6–8 MHz. The accesses were obtained on the common femoral artery (two patients left and one patient right), and long 5-French sheaths were advanced. Patients have given their consent for participation in this report.

### Case 1

**Fig. 3:**
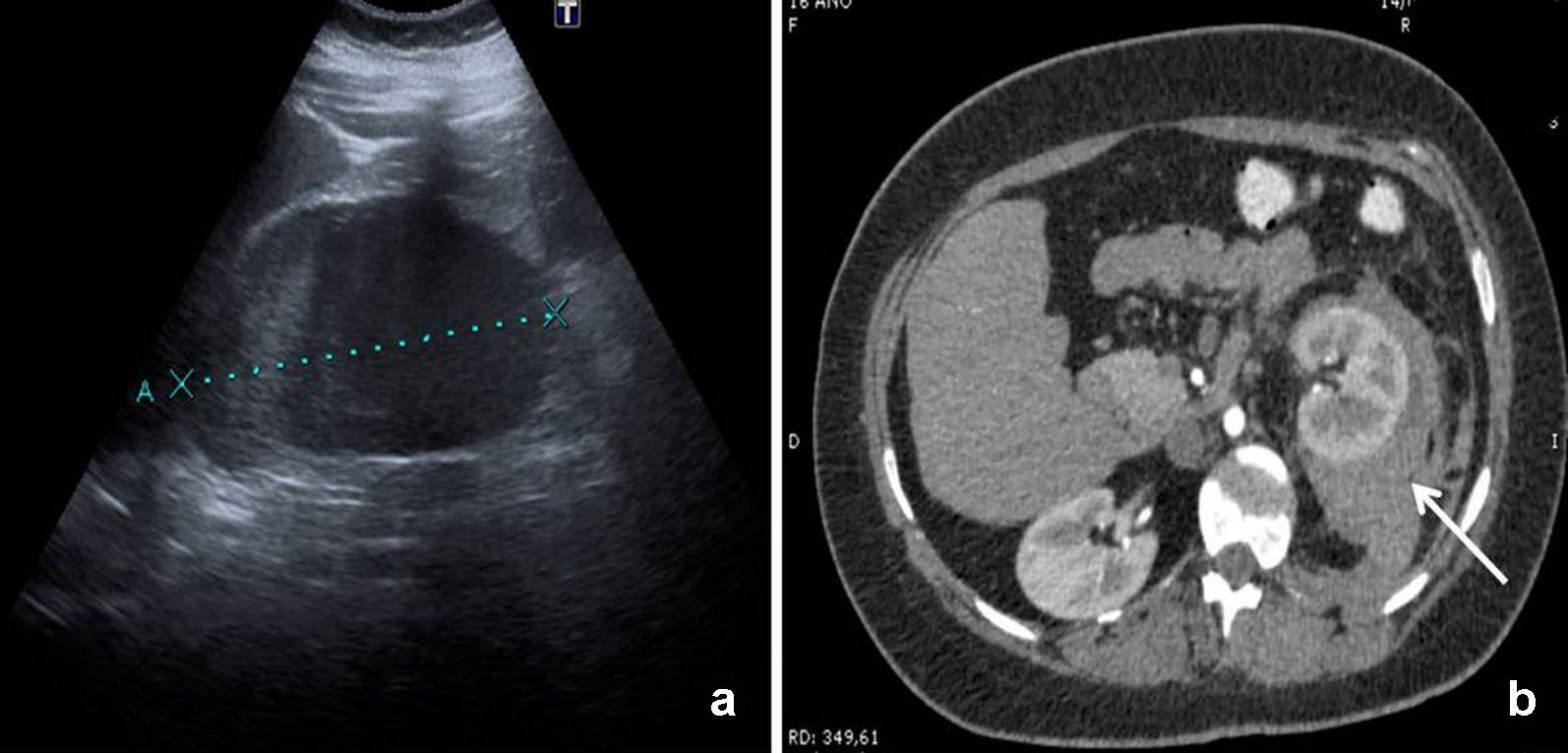
**a** r-EFAST: left perirenal hematoma of 1.5 cm (callipers). **b** CT Scan with intravenous contrast: peri- and pararenal hematoma (*arrow*) displacing anteriorly the left kidney

A 16-year-old woman with a previous medical history of partial atrioventricular canal defect and subpulmonary membrane, which had been surgically corrected when she was 5 years old, underwent a percutaneous stent implantation in the pulmonary artery due to a residual stenosis. The patient was taken to the Cardiology Service without experiencing any problems during the procedure. Within 24 h upon admission, the patient developed a progressive left flank pain with positive fist percussion. Within 48 h, she experienced a sudden clinical deterioration with hypotension and a decreased haemoglobin level (12.8–8.3 g/dl), and was subsequently transferred to the Intensive Care Unit (ICU). EFAST result was positive), revealing a small amount of fluid in the Douglas’ pouch. By performing r-EFAST, a left perirenal hematoma was found and identified as the likely origin of the hypotensive event. Abdominal CT scan with intravenous contrast revealed a left perirenal hematoma of 1.5 cms without signs of active contrast extravasations, with a small hematoma component open to the pararenal space (Fig. 3). The patient was successfully managed with conservative treatment. Serial r-EFAST showed no changes to the hematoma size or evidence of bleeding during the patient’s stay in ICU.

### Case 2

**Fig. 4:**
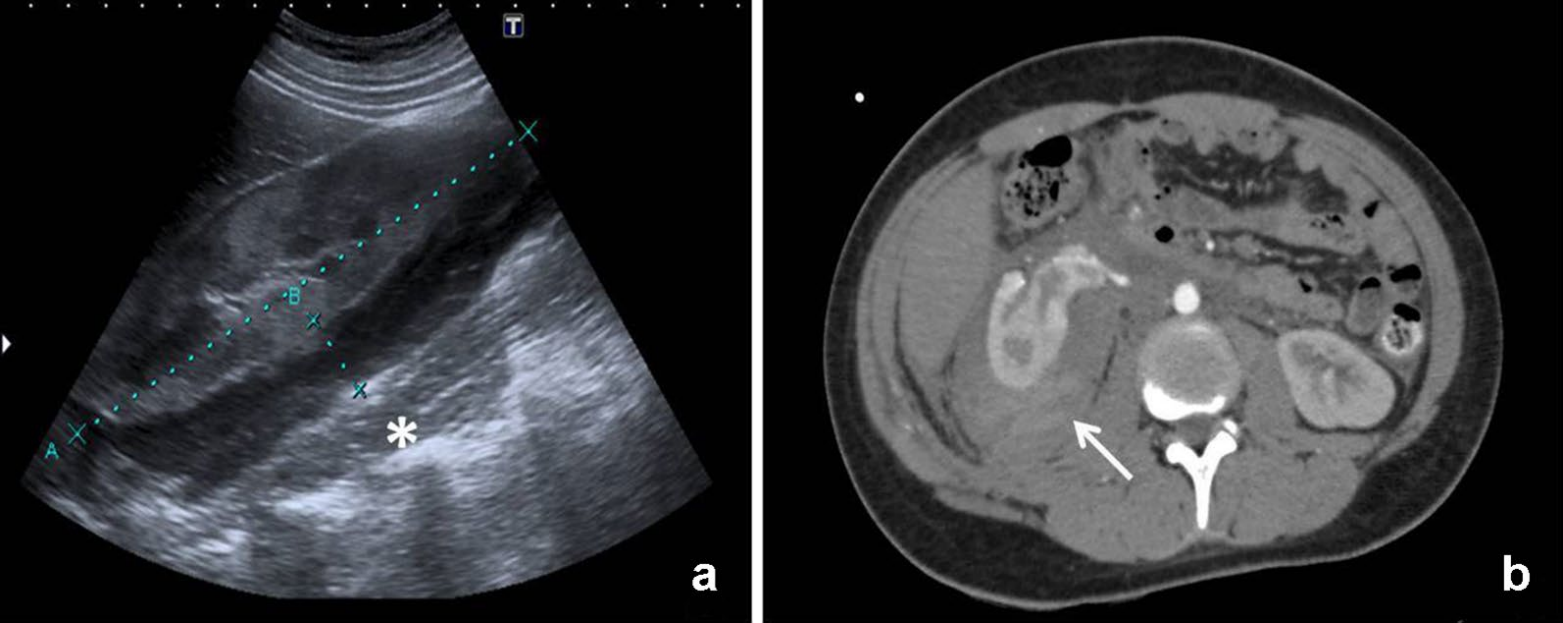
**a** r-EFAST: perirenal fluid collection anterior to psoas muscle (*asterisk*) measuring approximately 14 × 2 cms (callipers). **b** CT Scan with intravenous contrast: right perirenal hematoma with different densities that may correspond to active bleeding extending to pararenal space (*arrow*)

A 16-year-old male with a previous medical history of aortic coarctation and isthmus hypoplasia operated in the neonatal period. Due to recoarctation, cardiac catheterization (balloon angioplasty) was performed. Forty-eight hours after the procedure, he developed a sudden left flank pain, abdominal distension, palpitations, sweating, hypotension, tachycardia, obnubilation and decreasing levels of haemoglobin, resulting in ICU admission and transfusion of packed red blood cells. EFAST result was negative (no air in pleural space and absence of free fluid in the pleural, pericardial or intraperitoneal space). The r-EFAST findings were however consistent with retroperitoneal bleeding revealing a collection of fluid measuring 14 × 2 cm in the right perirenal space extending to pararenal, paravesical and presacral spaces. The abdominal—CT scan with intravenous contrast confirmed retroperitoneal US findings with no active bleeding signs; the intraperitoneal space was free of any fluid collection (Fig. 4). The patient showed no further signs of bleeding episodes and was discharged after 8 days.

### Case 3

**Fig. 5:**
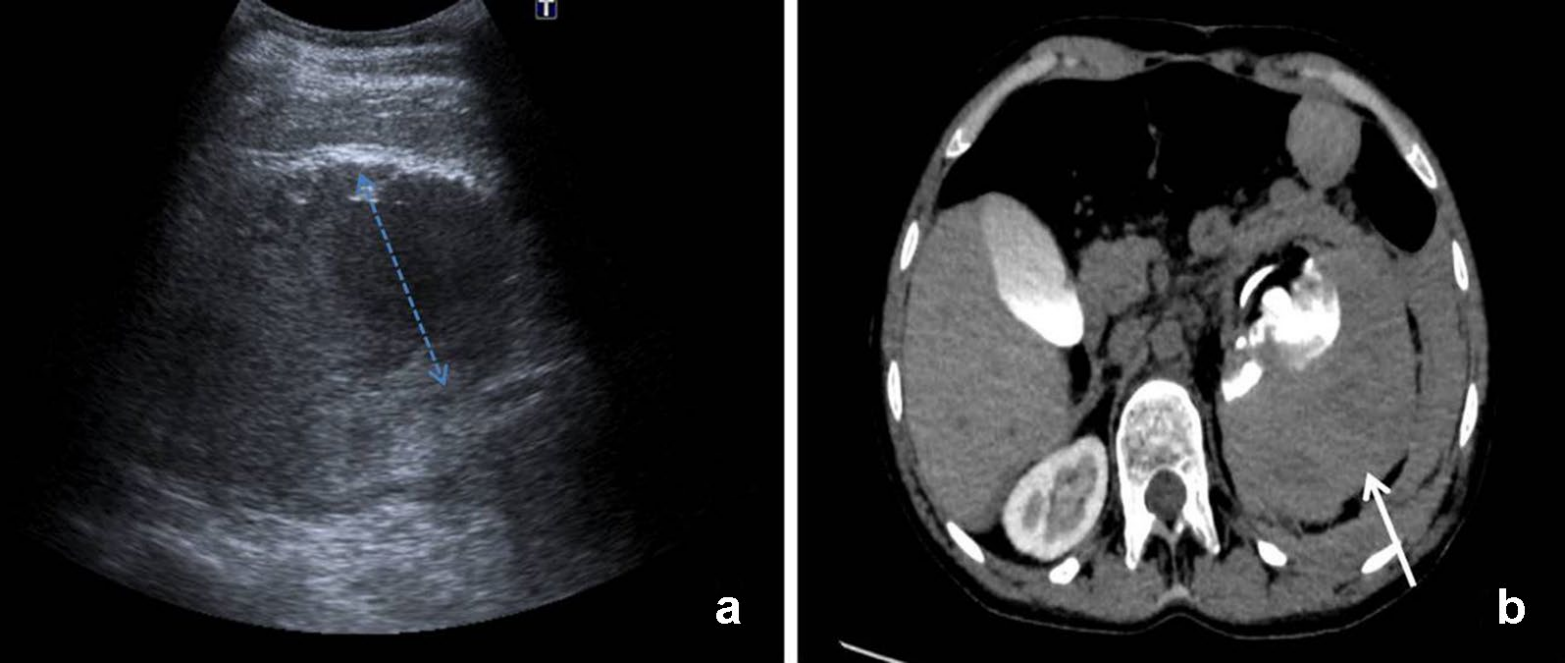
**a** r-EFAST: left renal hematoma of 6 cms (*arrow*). **b** CT Scan with intravenous contrast: perirenal hematoma (*arrow*), open to pararenal space. Also note the inhomogeneous distribution of intravenous contrast in the left kidney

A 21-year-old-woman with a diagnosis of tricuspid atresia, ventricular septal defect and transposition of great arteries in the Fontan stage, underwent a percutaneous cardiac procedure for stenting the Fontan conduit and the left pulmonary artery; no incidents were described during the procedure. After 6 h, the patient presented symptoms of diffuse abdominal pain and a decrease in haemoglobin level from 12 to 5 g/dl. EFAST resulted positive (intraperitoneal free fluid) and r-EFAST demonstrated a retroperitoneal hematoma confirmed with the abdominal CT scan with intravenous contrast (left renal hematoma up to 6 cms open to intraperitoneal space with multiple foci of active bleeding) (Fig. 5). A selective embolization of the left renal artery medial segment was performed but finally, due to the persistent hemodynamically instability, a nephrectomy was required.

## Conclusions

Standard EFAST can be performed by physicians as a non-invasive bedside tool. It was strictly designed for the rapid detection of free peritoneal/pericardial/pleural fluid and free pleural air benefitting from diagnostic accuracy without a significant inter-operator variability, having a medium sensitivity but high specificity. Point-of-care ultrasound is a significantly more powerful tool when performed in a high prevalence population. The design of new protocols is important in different pathologies and situations such as the case of EFAST in blunt trauma. EFAST has been implemented in other clinical scenarios and ultrasound protocols, such as shocked adult patients (RUSH exam) [9]. In a similar approach, Heller et al. [10] focused the assessment with sonography on human immunodeficiency virus co-infected tuberculosis patients (FASH protocol) to diagnose extra-pulmonary tuberculosis.

The modification in the EFAST technique, r-EFAST, permits the detection of organ injury and/or free fluids in the retroperitoneal space when complications are suspected, such as, hematoma perirenal or kidney injuries in hemodynamically unstable patients after percutaneous cardiac intervention with abdominal pain and/or decreased haemoglobin level. This event is previously suspected by clinical examination and eventually confirmed by bedside ultrasound.

The r-EFAST extends beyond the EFAST for detecting source of bleeding in the retroperitoneum, not contemplated in the original EFAST. Although r-EFAST may likely require additional procedural time in comparison with EFAST, it is highly dependent upon the experience of the physician (“a good FAST exam takes only 30 s”) [11]. We would therefore recommend including r-EFAST as part of training programmes for physicians who assist patients with congenital heart diseases.

Some limitations must be taken into account. Firstly, the reported series is small (*n* = 3). Secondly, all patients were hemodynamically unstable, so it must be assumed that the amount of bleeding was important, which could have improved the sensitivity of the protocol and partially compensate the inter-operator variability. In case of smaller losses of blood, the r-EFAST could fail. Further studies and researches in this field should be designed to establish the accuracy, sensitivity and specificity of the r-EFAST protocol.

## Abbreviations

FAST: Focused Assessment with Sonography for Trauma
EFAST: Extended Focused Assessment with Sonography for Trauma
r-EFAST: Modified Extended Focused Assessment with Sonography for Trauma
ICU: Intensive Care Unit
CT: computed tomography
US: ultrasound
